# Case Series: clinical challenges in pediatric plastic bronchitis

**DOI:** 10.3389/fped.2026.1715606

**Published:** 2026-04-02

**Authors:** Nouf Nasser Albalawi, Dina Alafandi, Ahmad Alessa, Ali Alqahtani, Yousef Alshehri, Haitham Almoffarreh, Ziyad Alkathiri

**Affiliations:** 1 King Abdullah Specialized Children’s Hospital, Riyadh, Saudi Arabia; 2 Ministry of National Guard Health Affairs, Riyadh, Saudi Arabia; 3 King Abdulaziz Medical City in Riyadh, Riyadh, Saudi Arabia; 4 King Abdullah Medical City, Mecca, Saudi Arabia; 5 Dr Sulaiman Al-Habib Medical Group, Riyadh, Saudi Arabia; 6 King Faisal Specialist Hospital & Research Centre, Riyadh, Saudi Arabia

**Keywords:** bronchial cast, congenital heart disease, Fontan procedure, pediatric, plastic bronchitis

## Abstract

**Background:**

Plastic bronchitis, also known as bronchial cast syndrome, is a rare but serious complication predominantly seen in children with congenital heart disease, particularly following Fontan-type surgeries. This disorder is characterized by the development of cohesive bronchial casts that may result in life-threatening obstruction of the airways.

**Methods:**

We present a case series of three pediatric patients with complex congenital heart disease who developed plastic bronchitis after Fontan-style palliative surgery. We detail the clinical presentation, diagnostic workup, management, and outcomes of these patients.

**Results:**

All three patients presented with acute respiratory symptoms and expectoration of bronchial casts. Each patient had previously undergone multiple prior cardiac surgeries, culminating in a Fontan-type procedure. Management of such patients focuses on symptomatic relief, supportive care, and, in some cases, bronchoscopy cast removal. Variable clinical courses and outcomes highlight the challenges in the diagnosis and management of this condition.

**Conclusion:**

Plastic bronchitis in children with congenital heart disease necessitates a high clinical suspicion and a multidisciplinary approach for prompt intervention. Further research is required to improve outcomes in this vulnerable population.

## 1 Introduction

Plastic bronchitis (PB) is a rare disease in children but a serious condition that requires rapid recognition and intervention. This condition most commonly affects children and is characterized by the formation of cohesive, branching casts that obstruct the distal and intermediate airways (1). These casts contain mucus and proteins that can potentially cause significant respiratory complications (2). Depending on the size of the casts produced, patients may present with various respiratory symptoms such as dyspnea, wheezing, pleuritic chest pain, or fever (1). Although surgical correction of congenital heart disease is the primary cause of PB, previously healthy children with a male predominance develop this condition (3). Despite identifying many other etiologies such as infections, autoimmune, and iatrogenic causes, patients with allergies or asthma are more susceptible to developing PB (1, 3). In 1997, researchers formalized and classified the histological patterns of PB, distinguishing two primary types of airway casts found in patients: type 1 inflammatory casts, composed of fibrin and acute-phase inflammatory cell infiltrates, and type 2 non-inflammatory acellular casts, composed of mucin (4). Subsequently, numerous classifications have been reported based on various criteria, including disease state, associations, and cast histology (5). The evolving classification of PB pathogenesis suggests that the disease process remains poorly understood. Considering these concerns, in this study, we present three pediatric cases to further explain the clinical spectrum, diagnostic challenges, and management approaches associated with PB in children.

## 2 Case 1

**Figure 1:**
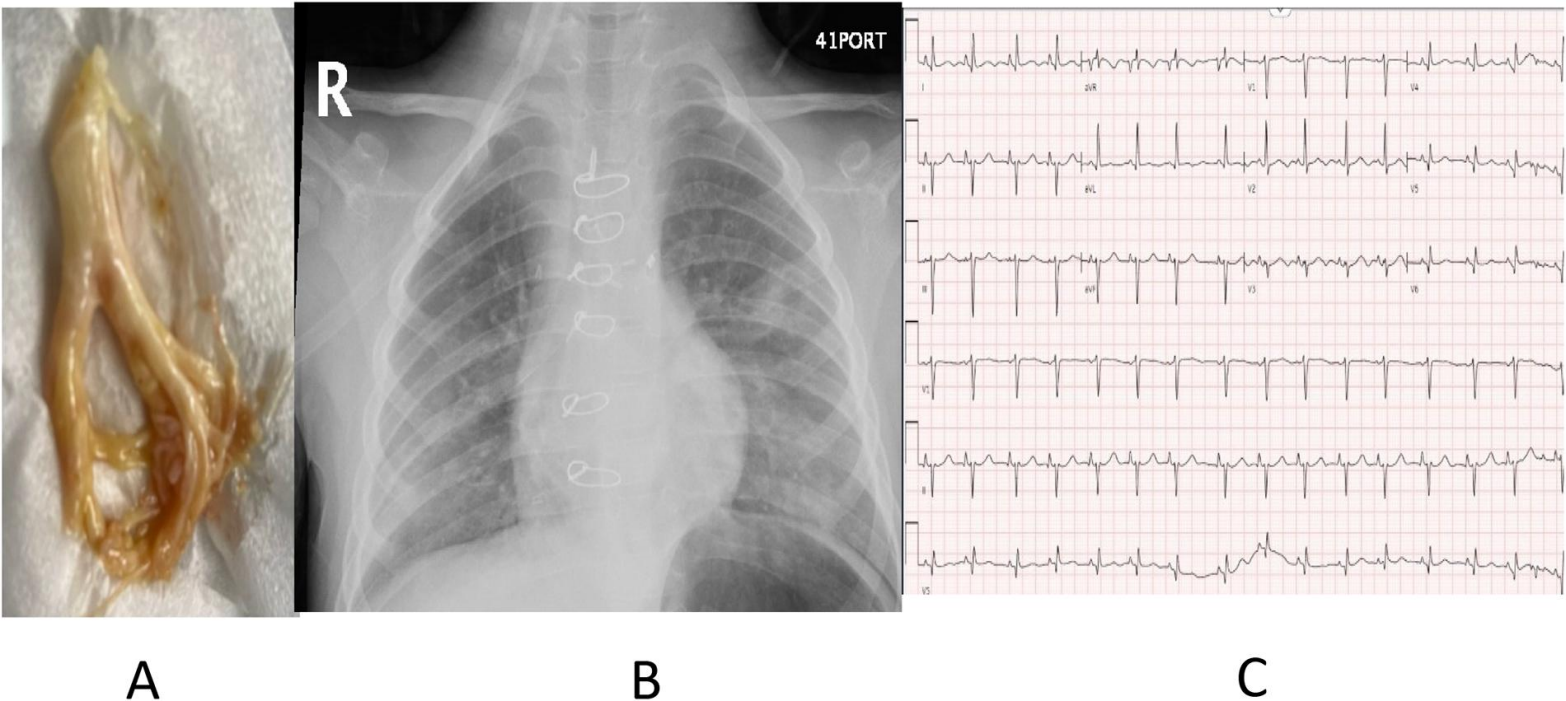
**(A)** White material expectorated by the patient suggestive of plastic bronchitis. **(B)** Chest radiograph (CXR) showed left mid and lower zone airspace opacities with perihilar peribronchial wall thickening. **(C)** Electrocardiogram (ECG) showed sinus rhythm with normal QRS andP-wave axes and generalized peaked P-waves related to the patient's atrial enlargement.

A 5-year-old boy presented with complex congenital heart disease, characterized by tricuspid atresia, with a severely hypoplastic right ventricle (RV), a hypoplastic pulmonary valve with small pulmonary artery (PA) branches, a small ventricular septal defect (VSD), and a narrow congenital abdominal aorta. His surgical history included modified Blalock–Taussig (BT) shunt placement and ligation of the patent ductus arteriosus in 2019. In the same year, he underwent a BT shunt with right pulmonary artery plasty, right- and left-sided bidirectional Glenn procedures, arterial septectomy, and ligation and division of the main pulmonary artery. Moreover, in 2023, he underwent an extracardiac fenestrated Fontan procedure. Our patient presented to the emergency department (ED) with a 3-day history of increased productive cough, spontaneous expectoration of white material (Figure 1A), fever, worsening respiratory symptoms, and desaturation. A physical examination revealed an oxygen saturation (SpO_2_) of 60%, prompting the use of oxygen to maintain an SpO_2_ target of 85% along with cyanosis, particularly perioral and acrocyanosis, mild diaphoresis, and diminished bilateral air entry and diffuse crepitation on auscultation of the chest. The initial chest radiograph (CXR) showed left mid- and lower-zone airspace opacities with perihilar peribronchial wall thickening, suggestive of small airway disease versus pneumonia (Figure 1B). An electrocardiogram (ECG) showed a sinus rhythm with normal QRS and P-wave axes and generalized peaked P-waves related to the patient's atrial enlargement (Figure 1C). On admission, he underwent cardiac catheterization (Table 1), which revealed a normal left ventricular end-diastolic pressure (LVEDP) of 8 mmHg and a mean Fontan pressure of 9 mmHg. Pulmonary vascular resistance was within the acceptable range, and the transpulmonary gradient (TPG) was 2 mmHg. The patient's hemodynamic data showed good diastolic function with normal Fontan pressure and TPG. An echocardiogram performed during admission showed a bilateral bidirectional superior cavopulmonary connection (Glenn anastomosis), total cavopulmonary connection Fontan circulation, laminar flow across the stent Fontan fenestration, good PA size with laminar flow, stented right pulmonary artery with laminar flow by a color-mean gradient of 2 mmHg, mild left atrioventricular (AV) valve regurgitation, unobstructed systemic outflow tract, good overall single ventricle systolic function, and no pericardial effusion. The patient was admitted to the pediatric cardiology ward, and the specimen was sent for histopathology, which showed few bronchial epithelial cells, pulmonary macrophages, scattered squamous cells, neutrophils, and lymphocytes. The background contained few fragments of degenerated material, admixed with inflammatory cells. A pediatric pulmonology team was involved in the treatment plan, who started the patient on nebulization such as aerosolized fibrinolytic agents, acetylcysteine 20%, steroids, 3% hypertonic saline, and systemic antibiotics, intravenous ceftriaxone and azithromycin. After a few days, the patient showed improvement and returned to his baseline discharge, with follow-up outpatient clinic visits with the pediatric cardiology and pulmonology teams. His discharge medications included inhaled steroids, aerosolized fibrinolytics, and a 3% hypertonic solution nebulizer.

**Table 1:** Case 1 cardiac catheterization results.

Parameter	Value	Unit
RFA pressure	105/60/79	mmHg
SVC pressure	10/10/9	mmHg
LV pressure	110/-/8	mmHg
LPA pressure	10/8/9	mmHg
IVC pressure	9/9/9	mmHg
RPA pressure	8/5/6	mmHg
PW pressure	7	mmHg
AO O_2_ saturation	99	%
SVC O_2_ saturation	75	%
IVC O_2_ saturation	75	%
LPA O_2_ saturation	72	%
CO	2.61	L/min
CI	3.7	L/min/m^2^
SVR	18.9	Wood units
PVR	0.61	Wood units

RFA, radio-frequency ablation; SVC, superior vena cava; LV, left ventricle; LPA, left pulmonary artery; IVC, inferior vena cava; RPA, right pulmonary artery; PW, pulse wave; AO, aorta; CO, cardiac output; CI, cardiac index; SVR, systemic vascular resistance; PVR, pulmonary vascular resistance.

## 3 Case 2

**Figure 2:**
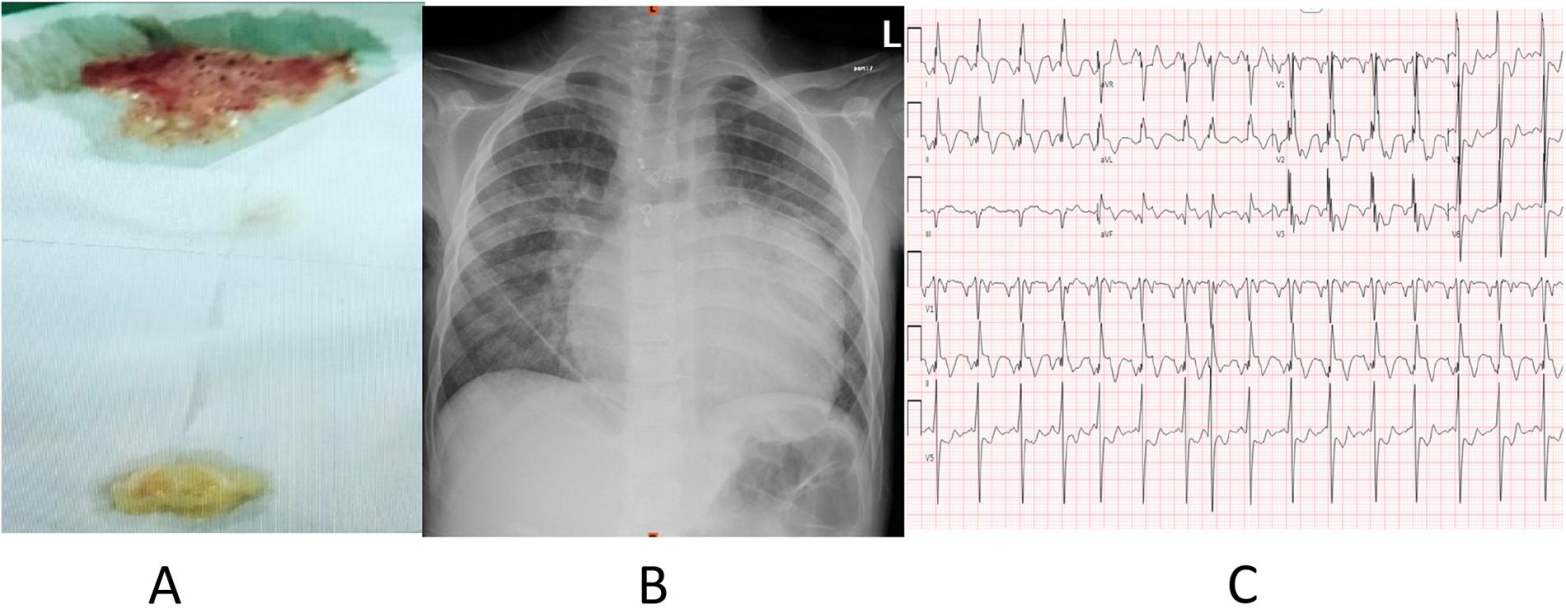
**(A)** Expectorated white material from the patient characteristic of plastic bronchitis. **(B)** Chest radiograph (CXR) showed the left lower lung showed opacities with silhouetting the left hemidiaphragm and left cardiac border associated with pleural effusion. **(C)** Electrocardiogram (ECG) showed an undetermined rhythm, non specific intraventricular conduction block, possible right ventricular hypertrophy, and ST elevation in the inferolateral leads (DII, DIII, aVF).

A 10-year-old boy presented with a history of a single ventricular pathway, characterized by a double-inlet left ventricle, a hypoplastic RV, L-malposed great arteries, a muscular ventricular septal defect, and a coarctation of the aorta. The patient presented to the ED with complaints of cough and respiratory distress for 3 days. His surgical history included multiple procedures in 2014, such as the Norwood procedure (the first-stage palliative surgery) and its associated condition, hypoplastic left heart syndrome, the bidirectional Glenn procedure, bilateral PA plasty, and right AV valve repair. The initial vital signs in the ED were normal, with an SpO_2_ of 51% on room air. A physical examination revealed that the patient was alert and cyanotic due to respiratory distress. A chest auscultation revealed decreased air entry, predominantly in the left hemithorax, with no added sounds. After the emergency team administered oxygen, the SpO_2_ increased to 89%. The initial CXR of the left lower lung showed opacities with silhouetting of the left hemidiaphragm and left cardiac border associated with pleural effusion (Figure 2B), and the ECG showed an undetermined rhythm, non-specific intraventricular conduction block, possible right ventricular hypertrophy, and ST elevation in the inferolateral leads (DII, DIII, and aVF) (Figure 2C). Upon admission, the patient coughed and spontaneously expectorated a material that appeared to be a plastic cast (Figure 2A). The patient underwent cardiac catheterization during admission, which revealed a normal LVEDP of 8 mmHg with a mean Fontan pressure of 11 mmHg. A hemodynamic testing of the patient revealed a satisfactory diastolic function, normal Fontan pressure, and normal TPG (Table 2). After being hospitalized in the pediatric cardiology ward, the patient was monitored by the pediatric pulmonology team and given symptomatic treatment such as nebulization: aerosolized fibrinolytic agents, acetylcysteine 20%, steroids, 3% hypertonic saline, and systemic antibiotics (intravenous meropenem and oral azithromycin). The patient recovered and resumed his baseline discharge after a few days with follow-up outpatient clinic visits with his primary and pulmonology teams, who continued inhaled steroids, aerosolized fibrinolytics, and 3% hypertonic solution nebulizer and salbutamol as home medications.

**Table 2:** Case 2 cardiac catheterization results.

Parameter	Value	Unit
AO pressure	81/45 (59)	mmHg
RPA pressure	14/10 (10)	mmHg
LPA pressure	14/11 (12)	mmHg
LV pressure	92/0 (8)	mmHg
PA O_2_ saturation	62	%
AO O_2_ saturation	76	%
SVC O_2_ saturation	54	%
IVC O_2_ saturation	54	%

AO, aorta; RPA, right pulmonary artery; LPA, left pulmonary artery; LV, left ventricle; PA, pulmonary artery; SVC, superior vena cava; IVC, inferior vena cava.

## 4 Case 3

**Figure 3:**
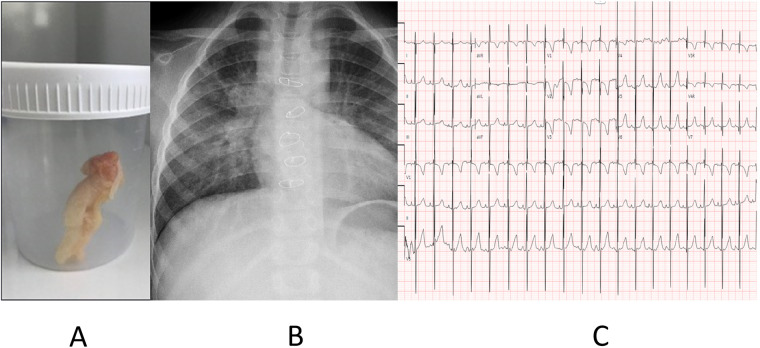
**(A)** A rubbery sputum which was expectorated by the patient. **(B)** Chest radiograph (CXR) demonstrated bilateral small airway disease with no focal consolidation. **(C)** Electrocardiogram (ECG) revealed a sinus rhythm with left axis deviation and a normal P wave axis.

A 10-year-old girl with a history of double-outlet RV, remote subpulmonary VSD, Taussig–Bing anomaly (a complex congenital heart defect characterized by a double outlet right ventricle and a subpulmonic VSD), subaortic systematic outflow obstruction, and chronic pericardial effusion presented to the ED. She experienced two episodes of cough with cyanosis, during which she spontaneously passed rubbery sputum mixed with mucus. Her surgical history included post-PA banding in 2014, post-Glenn and ligation and division of the main pulmonary artery in 2015, extracardiac fenestrated Fontan (18-mm Gore-Tex: expanded polytetrafluoroethylene), and atrial septectomy in 2017. On the primary survey, the girl’s vital signs were within the normal range, except for SpO_2_ (88%, acceptable for the patient's basal condition), and a chest auscultation revealed decreased air entry with bilateral crackles. An initial CXR revealed bilateral small airway disease with no focal consolidation (Figure 3B), and an ECG revealed a sinus rhythm with left-axis deviation and a normal P*-*wave axis (Figure 3C). During admission, the patient received symptomatic treatment such as nebulization: aerosolized fibrinolytics, acetylcysteine 20%, steroids, 3% hypertonic saline, and systemic antibiotics in the form of intravenous ceftriaxone. The patient passed a large, rubbery sputum sample (Figure 3A), which was sent for a histopathology examination that showed scattered inflammatory cells with negative cultures. Cardiac catheterization showed that the patient had a normal LVEDP, reflected by a wedge pressure of 7 mmHg and a mean Fontan pressure of 15 mmHg, with normal TPG. The patient's hemodynamic measurements revealed normal Fontan pressure and TPG, along with a satisfactory diastolic function (Table 3). The patient was observed and managed by the pediatric pulmonology team and treated for symptoms after admission to the pediatric cardiology unit. Finally, the patient recovered and started her baseline discharge with her primary team during a follow-up outpatient appointment.

**Table 3:** Case 3 cardiac catheterization results.

Parameter	Value	Unit
AO O_2_ saturation	92	%
DAO O_2_ saturation	92	%
LPA O_2_ saturation	64	%
IVC O_2_ saturation	45	%
RPA O_2_ saturation	65	%
SVC O_2_ saturation	65	%
LIV O_2_ saturation	71	%
PA O_2_ saturation	65	%
PV O_2_ saturation	92	%
AO pressure	99/49 (70)	mmHg
LPA pressure	15/13 (14)	mmHg
IVC pressure	15/15 (15)	mmHg
RPA pressure	15/14 (15)	mmHg
DAO pressure	95/48 (70)	mmHg
LV pressure	176/-	mmHg
PCW pressure	9/10 (7)	mmHg
RVOT pressure	72/39 (54)	mmHg
AAO pressure	101/52 (73)	mmHg

RFA, radio-frequency ablation; SVC, superior vena cava; LV, left ventricle; LPA, left pulmonary artery; IVC, inferior vena cava; RPA, right pulmonary artery; PW, pulse wave; AO, aorta; CO, cardiac output; CI, cardiac index; SVR, systemic vascular resistance; PVR, pulmonary vascular resistance.

## 5 Discussion

Various studies have provided guidance on how PB appears in both pediatric and adult populations, although its etiology remains unknown. Although numerous pathologies that cause PB, such as idiopathic, infectious, anatomic, surgical, and inflammatory processes, have been identified in emerging cases of this rare disease, only a few PB etiologies are common among the two populations (1, 6, 7). According to the literature, viral infections may result in the formation of bronchial casts. There have been reported cases of adenoviral and influenza B infections (3, 8, 9). There have been documented cases of bacterial infections, particularly *Mycoplasma pneumoniae*, *Pseudomonas paucimobilis*, *Staphylococcus aureus*, and group *Β* streptococci (10, 11). Moreover, there are numerous anatomical causes, including chyloptysis, thoracic duct stenosis, and lymph flow variations (12–14). In addition to surgical procedures, risk factors for PB include aortic arch reconstruction, postoperative chylothorax or ascites, extended chest tube drainage, and aortopulmonary collateral coiling (15).

PB is a known outcome of constrictive pericarditis and congenital heart diseases, including tricuspid atresia, tetralogy of Fallot, hypoplastic left heart syndrome, dextrocardia, single ventricle, total anomalous pulmonary venous return, and transposition of the great arteries. Postcardiac procedures include Fontan, Blalock–Taussig, and Glenn pulmonary arterioplasty; total caval pulmonary connections; atrioventricular valvuloplasty; right ventricular outflow tract restriction; and chylothorax (7, 16). Patients present to the emergency room with a history of fever, shortness of breath, cough, and acute and potentially fatal respiratory failure, which may mimic status asthmaticus or foreign body aspiration. Laboratory tests may show leukocytosis, and chest radiography may demonstrate atelectasis, infiltration, obstructive emphysema, bronchiectasis, pleural effusion, and pneumomediastinum. In one study, a physical examination revealed decreased breath sounds, wheezing, and dullness to percussion (7, 11). A high level of suspicion is necessary for the diagnosis of PB. Diagnosis is made by bronchoscopy and cast removal or by analyzing the cast after expectoration, although chest computed tomography is more advantageous (17).

PB management remains challenging and complex because of its rarity and varied causes (18). The only treatment for adult patients with idiopathic PB is cast removal (1). Several medical and surgical therapies have been developed for PB, reflecting advances in both supportive and interventional care. Pharmacological treatments are particularly useful for type I PB and include corticosteroids, macrolide antibiotics, sirolimus, N-acetylcysteine, heparin, plasminogen activators (urokinase and tissue plasminogen activator), DNase, and octreotide. However, bronchodilators, guaifenesin, and hypertonic saline have not demonstrated efficacy (1). Although the evidence for hypertonic saline in PB is limited and mixed, in our series, all three patients received 3% hypertonic saline as part of a multimodal regimen and showed clinical improvement, suggesting a potential adjunctive role that warrants further study.

Surgical and procedural approaches are more effective for type II PB, including extraction via bronchoscopy with cryoextraction or pretreatment with respiratory therapy to facilitate cast removal, lymphatic embolization to correct lymphatic abnormalities, thoracic duct ligation or stenting to manage lymphatic leaks, and lifestyle changes such as strict dietary fat restriction to reduce cast formation (1). Comprehensive respiratory care is crucial for managing PB during both acute and long-term phases. Airway clearance and pulmonary hygiene can reduce cast burden, prevent airway obstructions, and decrease respiratory complications (1, 5). Pulmonary hygiene strategies such as incentive spirometry, chest physiotherapy, postural drainage, percussion, vibration, and drainage techniques are widely recommended to enhance mucociliary clearance and reduce the risk of further cast formation or secondary infection (1).

In the case of our patients, 3% hypertonic saline was used alongside the aforementioned pulmonary hygiene measures and these may have contributed to improved airway clearance and symptom resolution. Furthermore, adjunctive therapies such as inhaled corticosteroids, bronchodilators, and mucolytics (e.g., dornase alfa) are tailored to the inflammatory or lymphatic nature of the disease. Inhaled tissue plasminogen activator and nebulized heparin have shown promise when conservative measures have failed (1, 5). In cardiac PB, especially with single-ventricle physiology, interventions such as lymphatic embolization and heart transplantation may be necessary (5). In addition, the use of overnight home high-flow nasal cannula (HFNC) therapy may prevent the recurrence of bronchial cast formation in children with PB. This is achieved by generating positive intra-airway pressure, which helps counter airway obstruction, while the delivery of warm, humidified air enhances mucociliary clearance and may soften airway secretions, thereby reducing bronchial cast formation (19). This approach suggests that HFNC may serve as an acute intervention and maintenance strategy in select patients. Moreover, PB continues to have a high recurrence rate and variable etiology and continues to pose therapeutic challenges. Patients with a history of complicated congenital cardiac disease were presented in our emergency room; each patient had previously undergone surgery, most notably versions of the Fontan technique. All patients exhibited signs of hypoxia, cyanosis, and respiratory distress. Finally, we established a collaborative multicenter study and defined treatment regimens to better understand and cure PB in the pediatric population.

## 6 Conclusion

Plastic bronchitis is a rare disease in children with congenital heart disease. A serious condition requires early recognition and a high index of suspicion. Prompt intervention depends on a coordinated multidisciplinary approach to airway management and stabilization. Despite advances in supportive care, outcomes remain variable and associated with significant morbidity. Further research is essential to define effective management strategies and improve long-term outcomes for this population.

## Data Availability

The original contributions presented in this study are included in the article/Supplementary Material, further inquiries can be directed to the corresponding author.
